# Head-to-head comparison of [^18^F]-Flortaucipir, [^18^F]-MK-6240 and [^18^F]-PI-2620 postmortem binding across the spectrum of neurodegenerative diseases

**DOI:** 10.1007/s00401-023-02672-z

**Published:** 2024-01-27

**Authors:** Cinthya Aguero, Maeva Dhaynaut, Ana C. Amaral, S.-H. Moon, Ramesh Neelamegam, Margaret Scapellato, Carlos Carazo-Casas, Sunny Kumar, Georges El Fakhri, Keith Johnson, Matthew P. Frosch, Marc D. Normandin, Teresa Gómez-Isla

**Affiliations:** 1 MassGeneral Institute for NeuroDegenerative Disease, Charlestown, MA USA; 2 Department of Neurology, Massachusetts General Hospital, WACC Suite 715, 15th Parkman St., Boston, MA 02114 USA; 3 Gordon Center for Medical Imaging, Department of Radiology, Massachusetts General Hospital, Boston, MA USA; 4 C.S. Kubik Laboratory for Neuropathology, Massachusetts General Hospital, Boston, MA USA

## Abstract

We and others have shown that [^18^F]-Flortaucipir, the most validated tau PET tracer thus far, binds with strong affinity to tau aggregates in Alzheimer's (AD) but has relatively low affinity for tau aggregates in non-AD tauopathies and exhibits off-target binding to neuromelanin- and melanin-containing cells, and to hemorrhages. Several second-generation tau tracers have been subsequently developed. [^18^F]-MK-6240 and [^18^F]-PI-2620 are the two that have garnered most attention. Our recent data indicated that the binding pattern of [^18^F]-MK-6240 closely parallels that of [^18^F]-Flortaucipir. The present study aimed at the direct comparison of the autoradiographic binding properties and off-target profile of [^18^F]-Flortaucipir, [^18^F]-MK-6240 and [^18^F]-PI-2620 in human tissue specimens, and their potential binding to monoamine oxidases (MAO). Phosphor-screen and high resolution autoradiographic patterns of the three tracers were studied in the same postmortem tissue material from AD and non-AD tauopathies, cerebral amyloid angiopathy, synucleopathies, transactive response DNA-binding protein 43 (TDP-43)-frontotemporal lobe degeneration and controls. Our results show that the three tracers show nearly identical autoradiographic binding profiles. They all strongly bind to neurofibrillary tangles in AD but do not seem to bind to a significant extent to tau aggregates in non-AD tauopathies pointing to their limited utility for the in vivo detection of non-AD tau lesions. None of them binds to lesions containing β-amyloid, α-synuclein or TDP-43 but they all show strong off-target binding to neuromelanin and melanin-containing cells, as well as weaker binding to areas of hemorrhage. The autoradiographic binding signals of the three tracers are only weakly displaced by competing concentrations of selective MAO-B inhibitor deprenyl but not by MAO-A inhibitor clorgyline suggesting that MAO enzymes do not appear to be a significant binding target of any of them. These findings provide relevant insights for the correct interpretation of the in vivo behavior of these three tau PET tracers.

## Introduction

In the past few years, important advances have been made in the tau neuroimaging field with the development of multiple novel PET tracers tailored to allow detection of tau pathology in the human living brain [2, 3, 9, 19]. This has opened the opportunity of using them as surrogate biomarkers to improve diagnostic accuracy in Alzheimer's disease (AD) and related tauopathies and to allow disease progression tracking in the human living brain. After a number of early failures, [^18^F]- Flortaucipir (also known as [^18^F]-T807, [^18^F]-AV-1451, and [^18^F]-Tauvid) was identified as the first promising tau PET tracer [7, 60]. Its utility for imaging tau in AD quickly became apparent [40, 45, 51], and this tracer gained FDA approval in 2020 to image tau pathology in patients being clinically evaluated for AD. We and others demonstrated that [^18^F]-Flortaucipir binds with strong affinity to tau aggregates in AD with a binding pattern that closely matches the accumulation and spreading of tau lesions through increasing Braak stages in parallel with the progression and severity of clinical symptoms of the disease [30, 32, 33, 37, 44]. But we also highlighted some limitations of this tracer, including its relatively low affinity for tau aggregates in non-AD tauopathies and its off-target binding to neuromelanin, melanin and blood products [25, 30–32, 46]. Several second-generation tau tracers have more recently been reported. The two that have gained significant attention are [^18^F]-MK-6240 [1, 18, 56] and [^18^F]-PI-2620 [5, 34, 39]; and both are now widely used in research settings [3, 5, 10, 27, 34]. We recently published our observations on the autoradiographic specificity and off-target binding patterns of [^18^F]-MK-6240 [1] on human postmortem tissue. Our data indicated that the binding behavior of this tracer closely parallels that exhibited by [^18^F]-Flortaucipir. [^18^F]-MK-6240 strongly binds to tangles in AD but does not seem to bind to a significant extent to tau aggregates in most non-AD tauopathies or to lesions containing β-amyloid, α-synuclein or TDP-43. Just like [^18^F]-Flortaucipir, [^18^F]-MK-6240 also exhibits strong off-target binding to neuromelanin and melanin-containing cells, and some weaker binding to areas of hemorrhage. Some controversy exists, with discrepancies among studies, as to whether [^18^F]-Flortaucipir and [^18^F]-MK-6240 may exhibit significant nonspecific binding to monoamino oxidase (MAO) enzymes [1, 3, 27, 36, 49], as it has been demonstrated for other tau PET tracers like THK-5351 [39]. This is something that needs to be settled because the correct identification of biological targets of imaging agents is an essential requirement for considering them as disease-specific and progression-specific biomarkers.

In the present study, we have had the opportunity to conduct a direct head-to-head comparison of the autoradiographic binding properties [^18^F]-Flortaucipir, [^18^F]-MK-6240 and [^18^F]-PI-2620 in the same postmortem brain tissue material from AD, non-AD tauopathies [Pick’s disease, progressive supranuclear palsy (PSP), corticobasal degeneration (CBD), chronic traumatic encephalopathy (CTE)], cerebral amyloid angiopathy (CAA), synucleinopathies (dementia with Lewy bodies (DLB)), transactive response DNA-binding protein 43 (TDP-43)-frontotemporal lobe degeneration (FTLD) and control cases. We have also conducted experiments to further investigate the potential non-specific binding of these three tracers to MAO enzymes. Our results indicate that these three tau PET tracers have nearly identical specific and off-target autoradiographic binding profiles and exhibit low binding activity for MAO enzymes.

## Material and methods

### Tissue samples

Postmortem brain, retina and skin tissue specimens from the Massachusetts and the Boston University Alzheimer’s Disease Research Centers Neuropathology cores were included in this study. Autopsies were performed according to standardized protocol [55]. Tissue collection and use was approved by the local Institutional Review Boards. A summary of the demographic characteristics and neuropathologic findings of the cases studied is shown in Table 1. Tissue material from several cases included in the present study was used in our previously published studies [1, 29, 31]. Additional tissue sections from those cases were prepared to run the new experiments presented here.

**Table 1:** Summary of the demographic characteristics of the postmortem brain samples studied

Case #	Pathological diagnosis	Age at death (years)	Gender	PMI (hours)	Braak and Braak (NFT)	CERAD score (neuritic plaques)
1	CTL	86	M	10	II	None
2	CTL	73	F	20	I	None
3	CTL	97	F	12	I	Sparse
4	AD	96	F	20	V	Frequent
5	AD	78	F	18	VI	Frequent
6	AD	87	F	12	IV	Moderate
7	AD	60	M	24	VI	Frequent
8	AD	82	F	6	V	Moderate
9	AD	69	F	4	VI	Frequent
10	AD	70	M	6	V	Frequent
11	AD	66	F	2	VI	Frequent
12	AD	66	F	10	VI	Frequent
13	AD	97	F	24	V	Frequent
14	AD	81	M	7	IV	Frequent
15	AD	101	F	22	II	Moderate
16	AD	66	M	16	V	Moderate
17	CAA (D23N Iowa APP mutation)	45	M	5	IV	Sparse
18	CTE (CTE stage III)	46	M	N/A	III	None
19	CTE (CTE stage IV)	65	M	N/A	II	Sparse
20	CTE (CTE stage III)	56	M	N/A	II	None
21	CTE (CTE stage II-III)	25	M	N/A	0	None
22	CTE (CTE stage III)	58	M	N/A	III	None
23	PiD	61	M	19	N/A	N/A
24	PiD	62	M	19	N/A	N/A
25	FTLD-TDP	55	M	14	I	None
26	FTLD-TDP	68	M	49	I	None
27	FTLD-TDP	64	M	12	I	None
28	FTLD-TDP	71	F	4	IV	None
29	FTLD-TDP	69	F	16	I	None
30	DLDH	65	M	24	I	None
31	PSP	69	M	45	N/A	None
32	PSP	68	M	48	N/A	None
33	PSP	78	M	11	N/A	None
34	PSP	73	M	12	N/A	None
35	PSP	63	F	12	N/A	None
36	PSP	78	F	4	N/A	None
37	PSP	63	F	34	N/A	Not assessed
38	CBD	80	M	6	N/A	None
39	LBD	62	M	24	N/A	Not assessed
40	LBD	76	M	17	II	Not assessed
41	LBD	83	M	9	III	None
42	MSA	60	F	32	II	None
43	ALS	70	M	14	I	None
44	Metastatic melanoma	75	M	35	V	Moderate
45	Subarachnoid Hemorrhage	92	F	16	V	Moderate

AD, Alzheimer’s disease; ALS, amyotrophic lateral sclerosis; CAA, cerebral amyloid angiopathy; CBD, corticobasal degeneration; CERAD, consortium to establish a registry for Alzheimer´s disease; CTL, control; DLDH, dementia lacking distinctive histopathology; F, female; FTLD, frontotemporal lobar degeneration; M, male; LBD, Lewy body disease; MSA, multiple system atrophy; N/A, not applicable; NFT, neurofibrillary tangles; PiD, pick’s disease; PSP, progressive supranuclear palsy; TDP, TDP-43

Histological evaluation of each case was routinely performed on a set of 19 blocked regions representative for a spectrum of neurodegenerative diseases. Paraffin-embedded blocks were stained with hematoxylin and eosin (H&E), Bielschowsky silver stain, and Aβ, phospho-tau, α-synuclein, ubiquitin and TDP-43 immunoreactivity. Blocks of frozen brain tissue containing hippocampal formation, entorhinal cortex (EC), frontal, parietal, temporal and occipital cortices, cingulate, cerebellum, basal ganglia and midbrain were cut into sections 10 μm-thick in a cryostat (Thermo-Shandon SME Cryostat), mounted on Histobond adhesion slides (StatLab, TX) and used for phosphor screen and nuclear emulsion high resolution autoradiography followed by immunohistochemistry using appropriate antibodies in each case.

### Phosphor screen autoradiography

[^18^F]-Flortaucipir, [^18^F]-MK-6240 and [^18^F]-PI-2620 were synthesized onsite as previously described [11, 20, 22, 47]. Autoradiography experiments were performed following our previously published protocols [1, 29, 31]. In brief, 10 μm-thick frozen sections were fixed at room temperature for 20 min in 100% methanol and then transferred to a bath containing high specific activity of the radiotracer in 10 mM PBS with a radioactivity concentration of approximately 20 μCi/ml (0.74 MBq/ml) for [^18^F]-Flortaucipir and 10 μCi/ml (0.37 MBq/ml) for [^18^F]-MK-6240 and [^18^F]-PI-2620.

Adjacent tissue slices were placed in a bath that was identical in all aspects except that the corresponding unlabeled tracer was added to yield 1 mM chemical concentration (for Flortaucipir and PI-2620) and 500 nM (for MK-6240), a blocking condition sufficient to saturate essentially all available specific binding sites of tau [60]. Additional adjacent slices were also incubated in separate baths containing either [^18^F]-Flortaucipir, [^18^F]-MK-6240 or [^18^F]-PI-2620 with a radioactivity concentration of approximately 20 μCi/ml, 10 μCi/ml and 10 μCi/ml, respectively, and selective MAO-A (clorgyline or harmine) and MAO-B (deprenyl) inhibitors (Sigma-Aldrich) were added at a competing concentration of 1 μM to assess potential displacement of the tracer binding signals. Slides were incubated for 60 min followed by a series of wash baths to remove unbound radiotracer. Wash solutions and incubation times were as follows: 10 mM PBS for 1 min, 70% ethanol/30% PBS for 2 min, 30% ethanol/70% PBS for 1 min, and 100% PBS for 1 min. Importantly, identical experiments were conducted in parallel but avoiding fixation in methanol as well as the use of ethanol in the wash solutions to rule out the possibility that that the use of methanol and/or ethanol could remove some weaker tracer binding in the autoradiography experiments. Slides were removed from the final wash solution and allowed to air dried before transferring them to a storage phosphor screen (FujiFilm BAS-IP MS2025E) that had been previously photobleached by exposure on a white light box for 15 min. The slides and phosphor screen were enclosed in an aluminum film cassette and left overnight in a dark room. Under dim lighting conditions, the screen was removed from the cassette and mounted to the digital biomolecular imager (Typhoon FLA 9000 storage phosphor imaging system). Scanning of screens was controlled by the Typhoon FLA 9000 Control Software using a resolution of 50 μm sampling interval. Digital images were saved in uncompressed form at full resolution and pixel depth. Images from adjacent brain slices incubated in the unblocked (high specific activity [^18^F]-Flortaucipir, [^18^F]-MK-6240 or [^18^F]-PI-2620) and blocking ([^18^F]-Flortaucipir plus 1 μM unlabeled Flortaucipir, [^18^F]-MK-6240 plus 500 nM unlabeled MK-6240 or [^18^F]-PI-2620 plus 1 μM unlabeled PI-2620) conditions were compared to determine total and non-specific binding in the tissue. All experiments were run in triplicates.

### High-resolution nuclear emulsion autoradiography and immunohistochemistry

To gain resolution at the cellular level, adjacent slides to those used in the phosphor screen autoradiography experiments, were dipped in a liquid photographic emulsion following our previously published protocols[1, 29, 31], followed by immunohistochemistry using the appropriate primary and secondary antibodies (primary antibodies used were: anti-tau PHF-1 (1:100, mouse, kind gift of Dr. Peter Davies), anti-Aβ (1:500, mouse, clone 6F/3D, Dako), anti α-synuclein (1:100, mouse, Zymed) and anti-phospho TDP-43 (pS409/410) (1:3000, mouse, Cosmo Bio CO); secondary antibodies used were ImmPRESS™ anti-mouse IgG (Vector Laboratories product MP-2400, Burlingame, CA) or ImmPRESS™ anti-rabbit Ig (Vector Laboratories product MP-7401, Burlingame, CA)) and developed with DAB solution (Vector Laboratories product SK-4100). H&E was used for counterstaining. Photomicrographs were obtained on an upright Olympus BX51 (Olympus, Denmark) microscope using visible light.

## Results

### [^18^F]-Flortaucipir, [^18^F]-MK-6240 and [^18^F]-PI-2620 phosphor screen autoradiography

**Fig. 1:**
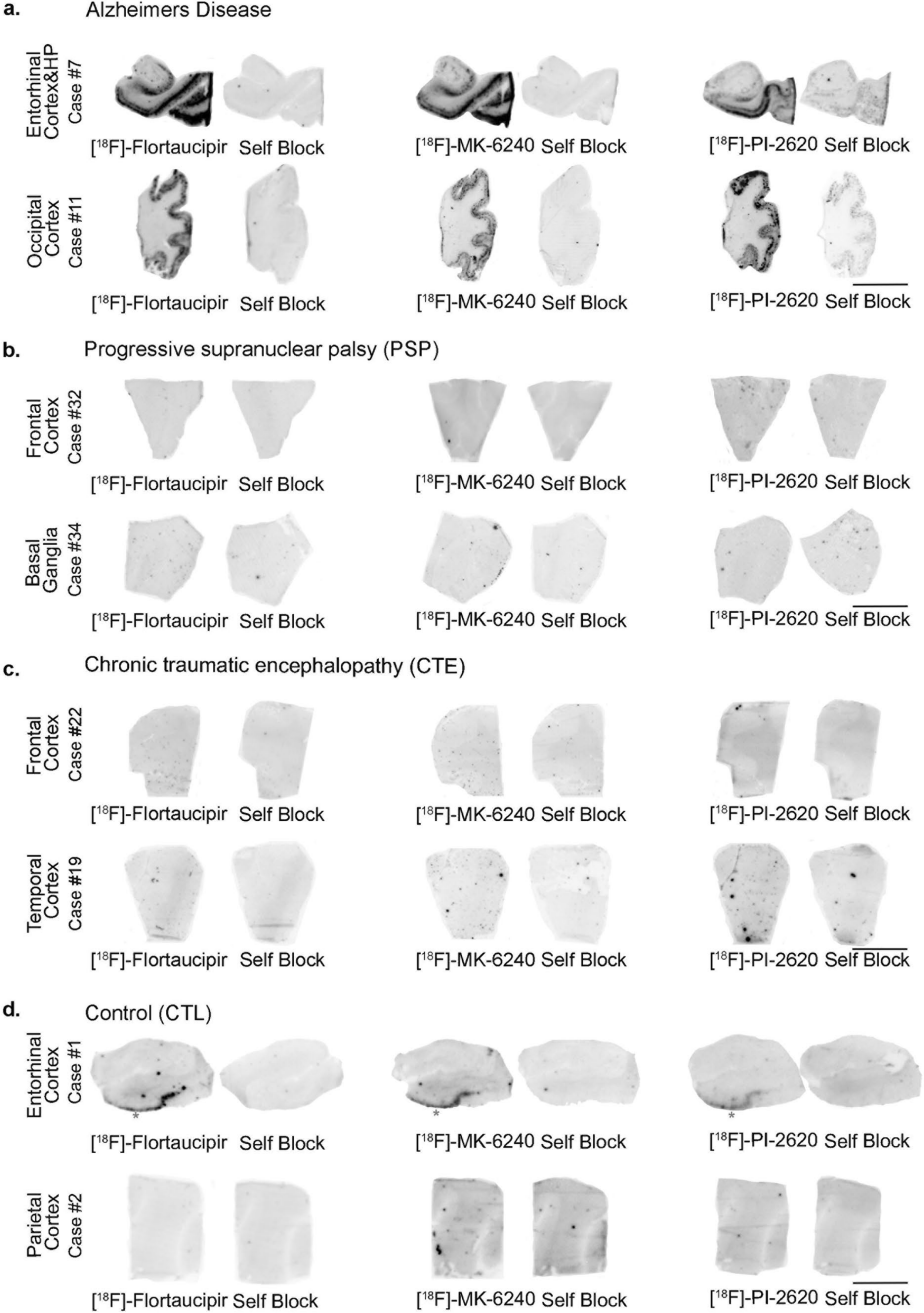
Representative images of [^18^F]-Flortaucipir (left), [^18^F]-MK-6240 (center), and [^18^F]-PI-2620 phosphor screen autoradiography of brain slices from AD (**a**), PSP (**b**), CTE (**c**) and control (**d**) cases. Strong binding of the three tracers was observed in cortical regions containing tangles (entorhinal cortex and occipital cortex) from AD brains. No signal was detected in regions containing tau aggregates in PSP (frontal cortex, basal ganglia) or CTE brains (frontal cortex, temporal cortex) or in control brains (parietal cortex) without pathology. An isolated binding signal of the three tracers was detected in the entorhinal cortex of the control case corresponding to the presence of age-related incidental tangles in that brain that nicely served as an internal positive control for the autoradiography experiments. The signal was blocked by adding the appropriate unlabeled tracer in each case. *Abbreviations* AD, Alzheimer disease; CTE, chronic traumatic encephalopathy; PSP, progressive supranuclear palsy. Scale bar = 1 cm

Phosphor screen autoradiography experiments demonstrated strong binding of [^18^F]-Flortaucipir, [^18^F]-MK-6240 and [^18^F]-PI-2620 in the hippocampal formation/entorhinal cortex (EC) and the neocortex from brain slices containing neurofibrillary tangles in AD cases (Fig. 1a). The binding signals were blocked after incubating the slides with the corresponding unlabeled tracer, demonstrating the selectivity of the signals. No binding signal was detected for any of the three tracers in regions lacking tangles in AD or in the control cases (Fig. 1d).

**Fig. 2:**
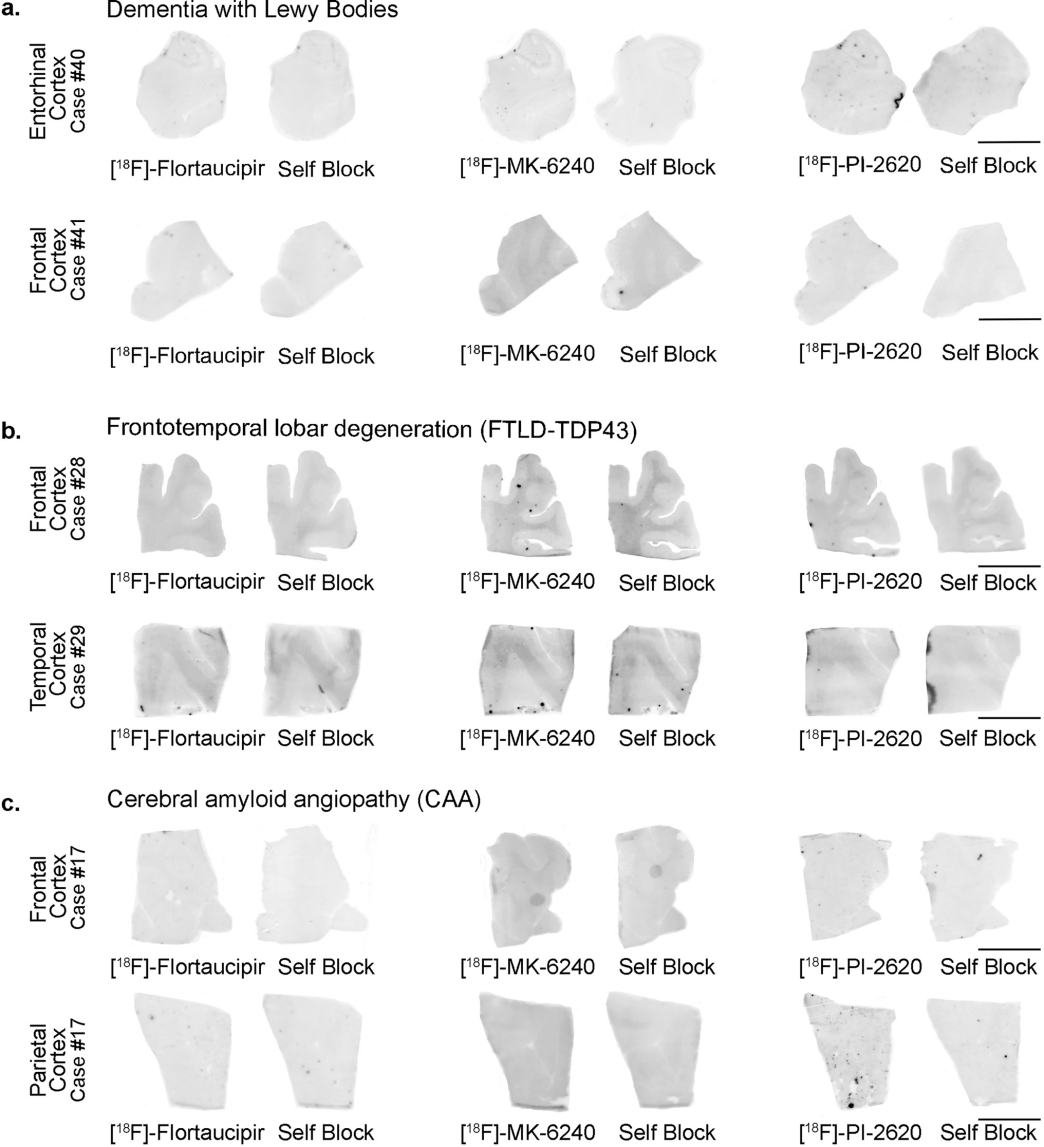
Representative images of [^18^F]-Flortaucipir (left), [^18^F]-MK-6240 (center), and [^18^F]-PI-2620 phosphor screen autoradiography of brain slices from DLB (**a**), FTLD-TDP-43 (**b**) and CAA (**c**). No binding of any of the three tracers was detected in slices containing Lewy bodies, TDP-43 inclusions or CAA lesions. *Abbreviations* CAA, amyloid angiopathy; DLB, dementia with Lewy bodies; FTLD-TDP-43, frontotemporal lobar degeneration-TAR DNA binding protein 43. Scale bar = 1 cm

**Fig. 3:**
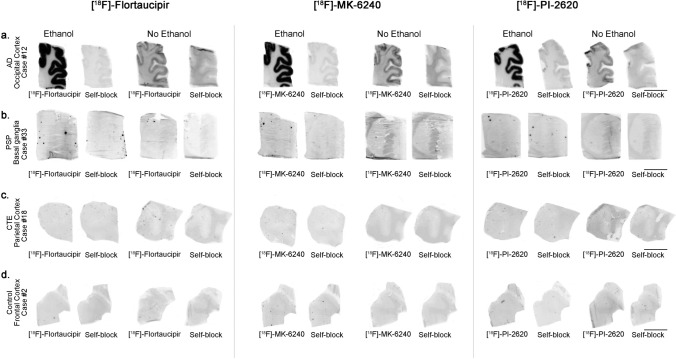
Representative images of [^18^F]-Flortaucipir (left), [^18^F]-MK-6240 (center), and [^18^F]-PI-2620 phosphor screen autoradiography experiments run in parallel with and without adding methanol/ethanol to the protocol washing steps. The parallel experiments yielded identical results: strong binding of the three tracers was observed in cortical regions containing tangles (occipital cortex) from AD brains. No signal was detected in regions containing tau aggregates in PSP (basal ganglia), CTE brains (parietal cortex) or in control brains (frontal cortex) without pathology. The signal was blocked by adding the appropriate unlabeled tracer in each case. *Abbreviations* AD, Alzheimer disease; CTE, chronic traumatic encephalopathy; PSP, progressive supranuclear palsy. Scale bar = 1 cm

**Fig. 4:**
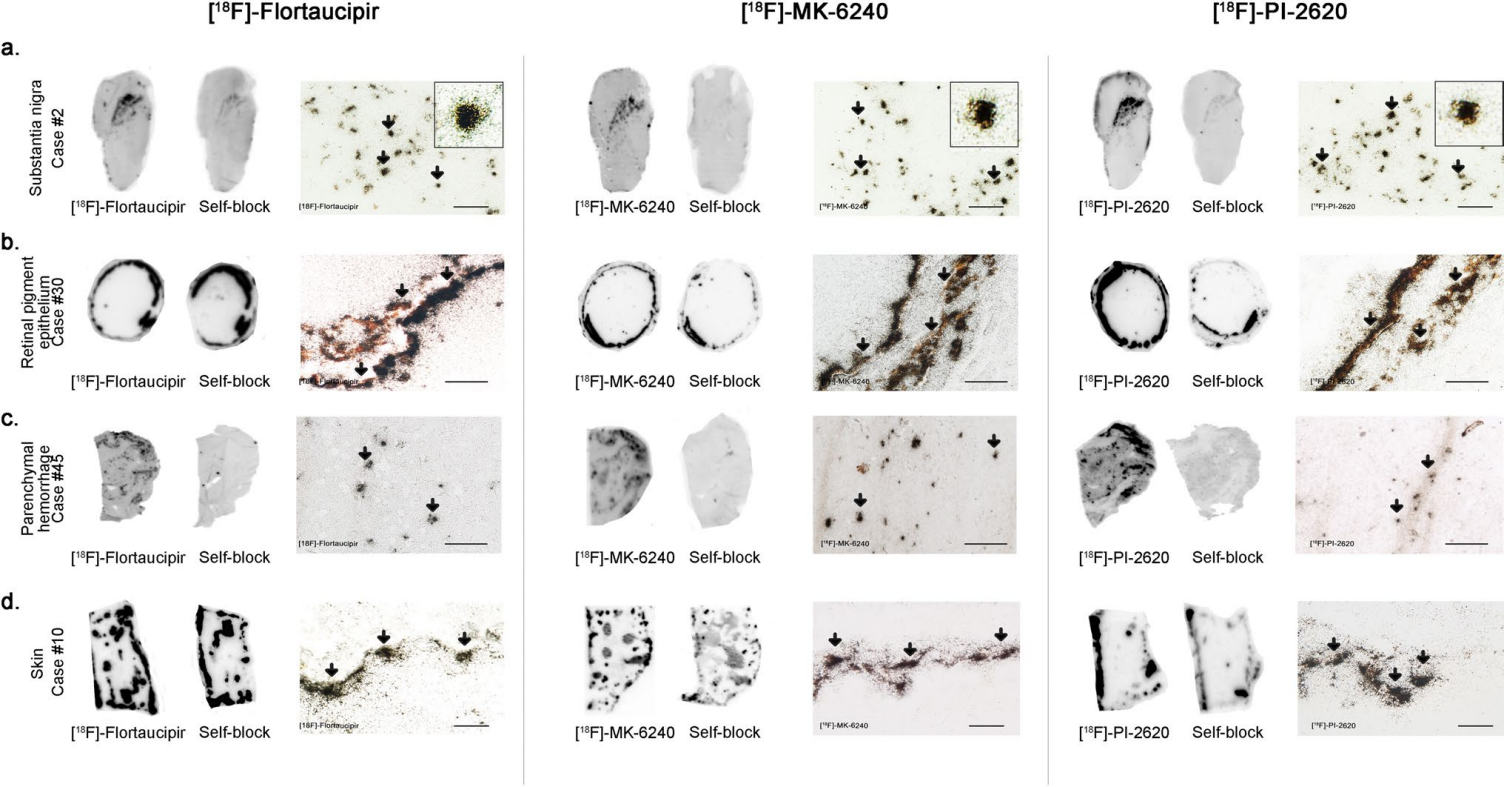
Representative images of [^18^F]-Flortaucipir (left), [^18^F]-MK-6240 (center), and [^18^F]-PI-2620 phosphor screen autoradiography experiments of slices containing substantia nigra in a control case (**a**), retinal pigment epithelium (**b**) in an dementia lacking distinctive histopathology case, parenchymal hemorrhagic lesions (**c**) and skin melanocytes (**d**) of an AD case. Strong binding of Flortaucipir, MK-6240 and PI-2620 was observed in neuromelanin-containing neurons of the substantia nigra (**a**), melanin containing granules in the retinal pigment epithelium (**b**), intraparenchymal hemorrhagic lesions (**c**) and skin melanocytes (**d**). Scale bars = 1 cm (**a**–**d** [^18^F]-Flortaucipir, [^18^F]-MK-6240 and [^18^F]-PI-2620 left panels: phosphor screen autoradiography) and 50 μm (**a**–**d** [^18^F]-Flortaucipir, [^18^F]-MK-6240 and [^18^F]-PI-2620 right panels; high resolution nuclear emulsion autoradiography)

**Fig. 5:**
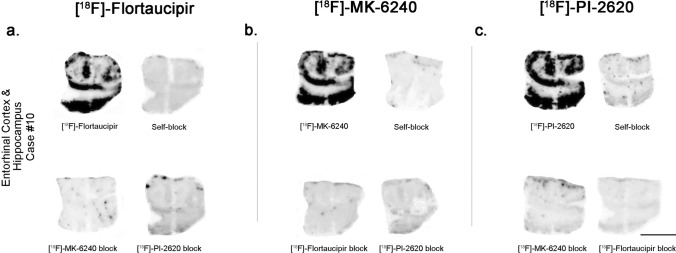
Head-to-head comparison of [^18^F]-Flortaucipir (left), [^18^F]-MK-6240 (center), and [^18^F]-PI-2620 phosphor screen autoradiographic binding patterns in adjacent sections obtained from the same tissue material containing entorhinal cortex from an AD case. The three tracers exhibited comparable strong binding to tangle-containing tissue material. Flortaucipir signal was almost completely blocked by adding 1 µM unlabeled MK-6240 or PI-2620; MK-6240 signal was almost completely blocked by adding 1 µM unlabeled Flortaucipir or PI-2620; and PI-2620 signal was almost completely blocked by adding 1 µM unlabeled Flortaucipir or MK-6240. Scale bar = 1 cm

No detectable [^18^F]-Flortaucipir, [^18^F]-MK-6240 or [^18^F]-PI-2620 binding could be observed in brain slices containing tau aggregates from Pick’s disease (not shown), PSP (Fig. 1b), CBD (not shown) and CTE (Fig. 1c). This is in agreement with previous observations by us and others [25, 30–32, 35, 46]**,** and favors the idea that these three tracers bind with significantly stronger affinity and selectivity to tau aggregates in AD but they all seem to have low affinity for tau aggregates in non-AD tauopathies. Brain slices containing TDP-43 inclusions, α-synuclein lesions, or CAA also lacked detectable Flortaucipir, MK-6240 or PI-2620 binding (Fig. 2a–c). Strong binding of the three tracers to incidental age-related neurofibrillary tangles that were present in the EC of some of these cases served as internal positive control (Fig. 2b). Ethanol-free parallel autoradiographic experiments yielded identical results, demonstrating that the ethanol washing steps didn’t remove some weaker tracer binding (Fig. 3a–d). Strong off-target binding signals of [^18^F]-Flortaucipir, [^18^F]-MK-6240 and [^18^F]-PI-2620 were detected in midbrain slices containing substantia nigra (Fig. 4a), regardless of the presence or absence of tau aggregates; as well as in the retinal pigment epithelium (Fig. 4b), brain slices containing parenchymal hemorrhages (Fig. 4c), and skin melanocytes (Fig. 4d), further favoring nearly identical autoradiographic specific and off-target binding patterns of the three tracers. Of note, [^18^F]-Flortaucipir, [^18^F]-MK-6240 and [^18^F]-PI-2620 could all be blocked by the other unlabeled compounds at 1 µM concentration (Fig. 5a–c).

**Fig. 6:**
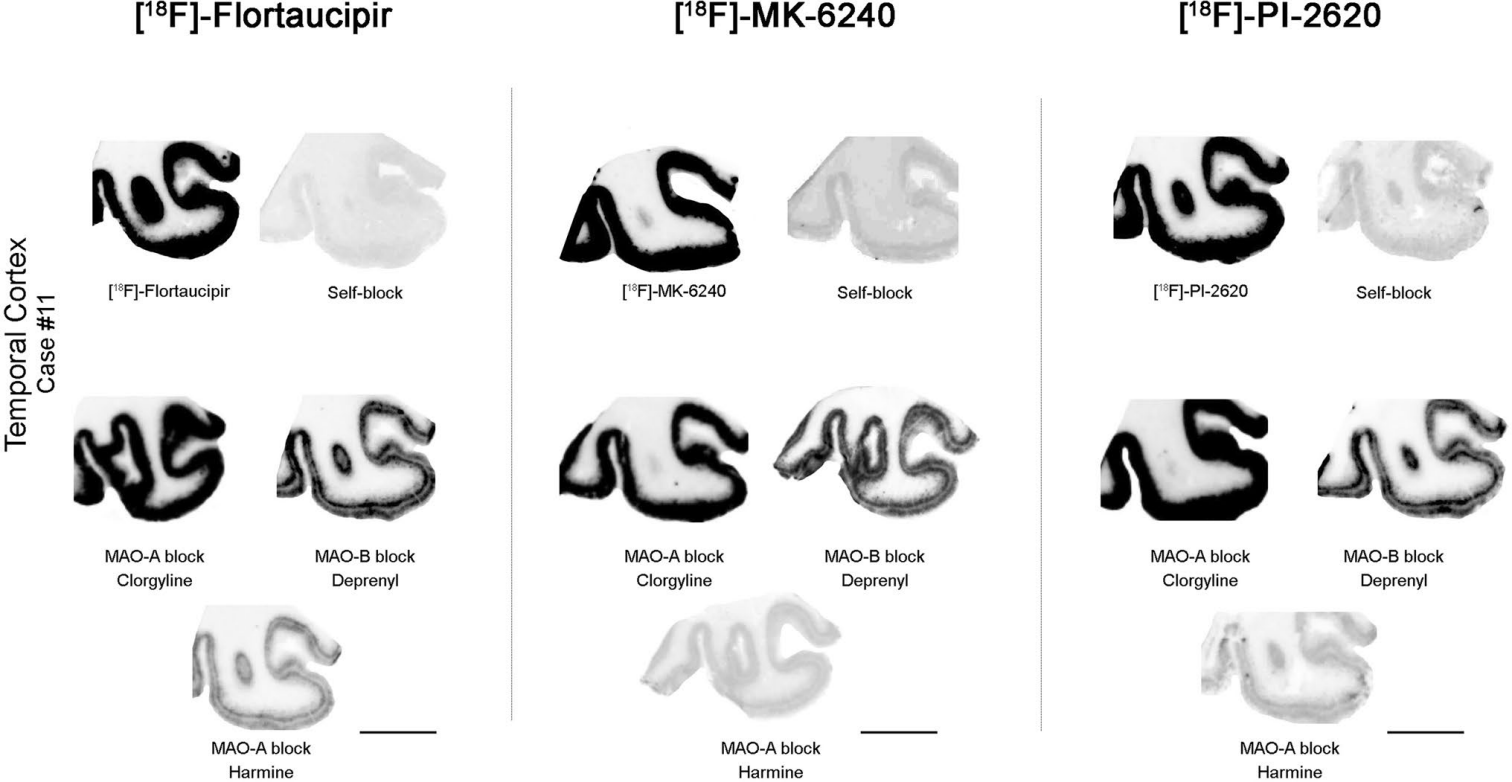
Representative [^18^F]-Flortaucipir (left), [^18^F]-MK-6240 (center), and [^18^F]-PI-2620 phosphor screen autoradiography experiments in adjacent slices containing temporal cortex from an AD case using competing concentrations of 1 μM clorgyline or harmine (MAO-A inhibitors) and deprenyl (MAO-B inhibitor). [^18^F]-Flortaucipir, [^18^F]-MK-6240, and [^18^F]-PI-2620 binding signals were only weakly displaced with 1 μM deprenyl (a selective MAO-B inhibitor). When a competing concentration of 1 μM clorgyline (a selective MAO-A inhibitor) was added to the blocking solution, no [^18^F]-Flortaucipir, [^18^F]-MK-6240 or [^18^F]-PI-2620 autoradiographic signal displacement could be detected. A more robust displacement of the three tracer signals was observed when a competing concentration of 1 μM harmine (dual inhibitor of DYRK1A and MAO-A) was added to the blocking solution. Scale bar = 1 cm

When a competing concentration of 1 μM clorgyline (a selective MAO-A inhibitor) was added to the blocking solution, neither [^18^F]-MK-6240, [^18^F]-Flortaucipir or [^18^F]-PI-2620 autoradiographic signals were displaced (Fig. 6). Only a weak displacement of the three tracer binding signals was noted when a competing concentration of 1 μM deprenyl (a selective MAO-B inhibitor) was added to the blocking solution (Fig. 6) suggesting that neither MAO-A or B are relevant binding affinity sites of any of these three tracers. Interestingly though, a more robust displacement of the binding signals of the three tracers was noted when adding 1 μM harmine (another reversible inhibitor selective for MAO-A that is also known to inhibit protein kinase DYRK1A and tau phosphorylation at multiple AD-related sites [16]) to the blocking solution (Fig. 6); our interpretation of this result is discussed further below.

### [^18^F]-Flortaucipir, [^18^F]-MK-6240 and [^18^F]-PI-2620 high resolution nuclear emulsion autoradiography and immunohistochemistry

**Fig. 7:**
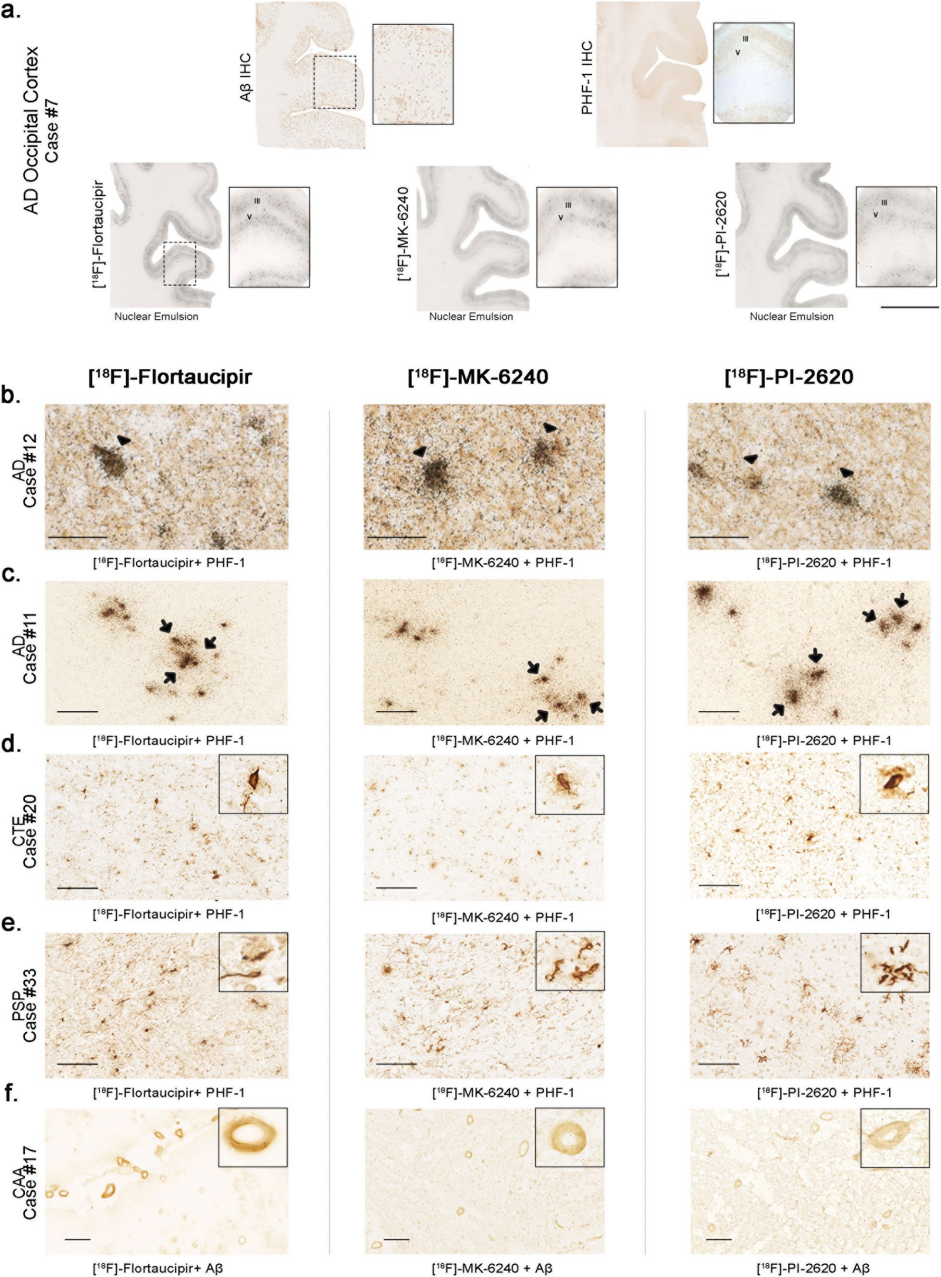
Representative [^18^F]-Flortaucipir (left), [^18^F]-MK-6240 (center), and [^18^F]-PI-2620 (right) high resolution autoradiography photomicrographs of brain slices containing occipital cortex from AD (**a**, **b**), temporal cortex from CTE (**c**), basal ganglia from PSP (**d**) and occipital cortex from CAA (**e**) and immunostaining with appropriate antibodies [PHF-1 antibody (kind gift of Dr. Peter Davies) and anti-Aβ antibody (1:500, mouse, clone 6F/3D, Dako)]. High resolution nuclear emulsion autoradiography showed a strong cortical accumulation of silver grains with the three tracers in cortical layers III and V (**a**) in AD brains mirroring the laminar pattern of tangles on adjacent slices as revealed by PHF-1 immunostaining rather than the more scattered plaque distribution pattern revealed by Aβ immunostaining (**a**). Strong silver grain accumulation was observed coinciding with the location of phosphor-tau immunoreactive cell somas (arrowheads) (**b**) and neuritic dystrophies (arrows) (**c**) in AD. No silver grains could be detected co-localizing with any of the three tracers in tau aggregates in CTE (**d**) or PSP (**e**) or with vascular amyloid deposits in CAA (**f**). *Abbreviations* AD, Alzheimer disease; CAA, amyloid angiopathy; CTE, chronic traumatic encephalopathy; PSP, progressive supranuclear palsy. IHC, immunohistochemistry. Scale bars = 1 cm (**a**), 50 μm (**b**), 100 μm (**c**–**e**), 200 μm (**f**)

Dipping adjacent brain slices to those used in phosphor screen autoradiography in a photographic nuclear emulsion, we were able to visualize silver grains struck by positrons emitted during [^18^F] nuclear decay and thus precisely identified [^18^F]-Flortaucipir, [^18^F]-MK-6240 and [^18^F]-PI-2620 labeled lesions by optical microscopy. We confirmed the presence of strong and selective concentrations of silver grains in tissue sections from AD cases, reflecting underlying [^18^F]-Flortaucipir, [^18^F]-MK-6240 and [^18^F]-PI-2620 binding of these three tracers to neurofibrillary tangle containing regions in AD brains that closely matched the laminar distribution of tangles on adjacent slices as revealed by PHF-1 immunostaining but not the plaque distribution pattern revealed by Aβ immunostaining (Fig. 7a). [^18^F]-Flortaucipir, [^18^F]-MK-6240 and [^18^F]-PI-2620 high resolution autoradiography followed by immunostaining with PHF-1 or Aβ antibodies on the same brain slices confirmed that the lesions labeled by the nuclear emulsion were tau aggregates, including classic AD tau tangles (Fig. 7b) and tau containing dystrophic neurites (Fig. 7c), but not Aβ plaques themselves or vessels with β-amyloid deposits (Fig. 7f). Tangle-containing slices from AD brains dipped in the nuclear photographic emulsion omitting the incubation with each tracer showed no silver grain accumulation and served as negative control (not shown). Negligible numbers of silver grains were observed colocalizing with tau aggregates in non-AD tauopathy cases (Fig. 7d, e), and no silver grains were observed either colocalizing with α-synuclein or TDP-43 containing inclusions (not shown). In agreement with the phosphor screen results above, neuromelanin-containing neurons in the substantia nigra (Fig. 4a), retinal pigment epithelium (RPE) cells (Fig. 4b), and skin melanocytes (Fig. 4d), consistently demonstrated robust concentration of silver grains confirming off-target binding of the three tracers to neuromelanin- and melanin-containing cells. Weaker concentrations of silver grains were also observed colocalizing with parenchymal hemorrhages (Fig. 4c), confirming additional off-target binding of the three tracers to blood products.

## Discussion

Validating the underlying neuropathological binding substrates and identifying potential off-target binding of tau PET ligands is critical for the accurate interpretation of their in vivo imaging behavior. In the present study, we conducted a direct head-to-head comparison of the autoradiographic binding properties of the three most widely used tau PET ligands in clinical and research studies, [^18^F]-Flortaucipir, [^18^F]-MK-6240 and [^18^F]-PI-2620, in a collection of postmortem tissue samples representing a broad spectrum of neurodegenerative disorders. Our main goal was to identify similarities and potential differences in their specific binding properties and off-target profiles by performing combined sensitive autoradiography and immunohistochemistry experiments in the same tissue material, using these three compounds at similar concentrations used in vivo for PET studies. Investigating whether any of these three tracers may exhibit a more advantageous autoradiographic binding profile towards the goal of accurately detecting and tracking progression of tau pathology in the human living brain is relevant for the eventual implementation of the second-generation tau tracers in clinical practice and may also help guiding tracer comparison studies of in vivo data that are currently being collected across different institutions in research settings.

Our results demonstrate that [^18^F]-Flortaucipir, [^18^F]-MK-6240 and [^18^F]-PI-2620 have a similar high binding affinity for tau aggregates in AD brain tissue, but none of them seems to bind to a significant extent to tau aggregates in non-AD tauopathies such as PiD, PSP, CBD or CTE, or to Aβ, α-synuclein or TDP-43-containing lesions. The laminar and cellular autoradiographic patterns of distribution of [^18^F]-Flortaucipir, [^18^F]-MK-6240 and [^18^F]-PI-2620, as revealed by the combination of autoradiography using a fine grain nuclear emulsion and immunohistochemistry, are also very similar across tracers and closely match those of classic PHF-tangles in AD brains. In agreement with our previously published results on [^18^F]-Flortaucipir and [^18^F]-MK-6240 [1, 29, 31, 33], nuclear emulsion dipped slides after incubation with each tracer exhibited a very similar pattern at the cellular level for the three tracers, with high concentrations of silver grains coinciding with the typical location of tau aggregates in neuronal somas and swollen neuritic processes in AD brains. This suggests that neurofibrillary tangles and dystrophic neurites (predominantly clustered in the pattern of neuritic plaques) are the likely major pathological substrates of Flortaucipir, MK-6240 and PI-2620.

The microscopic examination of tau lesions in non-AD tauopathy brain samples confirmed the absence of detectable accumulation of silver grains of the photographic emulsion associated with those lesions for any of the three tracers. These observations further favor the idea that tau in tangles and dystrophic neurites of AD has a unique conformation, different from tau in neuronal and glial aggregates in non-AD tauopathies—as recently demonstrated by crioEM studies [12–14, 61]—that is recognized by these three tau ligands. This is consistent with the selection process that was used for their development as leading imaging agents using homogenates from AD tissue rich in NFT as the binding target [18, 20, 34, 60]. We cannot rule out the possibility that these tracers might exhibit different binding affinity depending on the maturity of the tau lesions, as it has been suggested by others [25], since all AD cases included in the present study, except for one, were at high Braak tangle stages (IV-VI) (Table 1). Our data also establish that Flortaucipir, MK-6240 and PI-2620 are not fully selective for PHF tau deposits. The three tracers share a very similar off-target profile and exhibit a strong non-specific binding to neuromelanin, melanin, and to a weaker extent to brain hemorrhagic lesions. In agreement with previous observations by us and others [1, 20, 30, 32], [^18^F]-Flortaucipir, [^18^F]-MK-6240 and [^18^F]-PI-2620 binding signals are only weakly displaced using autoradiography competition with unlabeled selective MAO-B inhibitor deprenyl, and not displaced at all by selective MAO-A inhibitor clorgyline, suggesting that none of the three tracers has a substantial binding affinity for MAO enzymes in the human brain.

An increasing amount of data collected from human in vivo [^18^F]-Flortaucipir, [^18^F]-MK-6240 and [^18^F]-PI-2620 PET imaging studies have been published [19, 34, 42, 43]**.** Results from the different studies have consistently shown an excellent signal-to-noise ratio of each of these three tracers for imaging brain tau deposition in AD with a several fold higher in vivo retention in target regions consistent with neuropathological neurofibrillary tau staging in AD patients compared to cognitively unimpaired individuals [3, 4, 6, 15, 19, 34, 41, 42]. Moreover, recent in vivo head-to-head comparison studies of [^18^F]-Flortaucipir, [^18^F]-MK-6240 have shown very similar retention profiles of both tracers and comparable diagnostic performance to discriminate patients with AD from cognitively unimpaired individuals [17, 21, 49]. This is in concordance with the present results as well as with previous confirmatory autoradiography studies conducted on brain samples representative of the spectrum of neurofibrillary tangle Braak staging (I–VI) in AD [25, 30, 46]. By contrast, the majority of [^18^F]-Flortaucipir and [^18^F]-MK-6240 in vivo imaging studies conducted in patients with frontotemporal lobal degeneration (FTLD) and predicted underlying tauopathy or TDP-43 pathology have demonstrated limited sensitivity and specificity of these two tracers in these conditions [22, 28, 52] with the notable exception of patients harboring the R406W mutation in the *MAPT* gene, encoding tau [23, 48, 58]; a mutation that results in FTLD with parkinsonism with a clinical phenotype resembling AD and abundant numbers of 3- and 4-repeat tau aggregates with a pair helical filament ultrastructure similar to neurofibrillary tangles found in AD [24]. These findings are in agreement with the present study and our previous postmortem validation analyses that demonstrated that [^18^F]-Flortaucipir and [^18^F]-MK-6240 have very similar binding affinity profiles in AD (high afflinity) vs. non-AD tauopathies (low affinity) [1, 30, 31]. Importantly, detailed clinic-pathologic correlation studies in patients with non-AD tauopathies who underwent [^18^F]-Flortaucipir PET scans while alive and came to autopsy have further shown the lack of a consistent and robust correlation between levels of in vivo tracer retention and the topographical distribution and burdens of tau lesions quantified at postmortem [15, 26, 32]. Clinico-pathologic correlation studies of MK-6240 in non-AD tauopathy cases are still lacking at this time. Current controversy exists over whether PI-2620 tracer may exhibit a more favorable binding affinity profile to 4-repeat tau aggregates in PSP and CBD in comparison to Flortaucipir and MK-6240. A recent study using post-mortem radioligand binding studies and autoradiography comparing [^3^H]-PI-2620, [^3^H]-MK-6240 and [^3^H]-RO948 concluded that those three tracers displayed a similar binding behavior in AD brains (in both homogenate competitive studies and one large frozen hemispherical brain section autoradiography studies) while autoradiography studies in the frontal cortex of CBD and PSP brains showed high specificity for [^3^H]-PI-2620 but not for [^3^H]-MK-6240 or [^3^H]-RO948 [27]. A multicenter cross-sectional PET imaging study on 60 patients with clinically suspected PSP found a significant elevation of [^18^F]-PI-2620 retention in PSP target regions with strongest differences in distribution volume ratio (DVR) values in PSP vs control groups in the globus pallidus internus (1.21 ± 0.10 vs. 1 ± 0.08) [5]. Surprisingly though, no significant association was found in that study between level of [^18^F]-PI-2620 in vivo retention and disease duration or clinical severity of symptoms arguing against the reliability of this tracer to accurately detect and track the expected progressive accumulation of tau pathology in PSP. Further radiological-pathological studies on individuals with pathologically-confirmed PSP at autopsy and available antemortem [^18^F]-PI-2620 imaging data seem necessary to resolve these apparent discrepancies. In the present study, we investigated the possibility that the discrepancies between the above [^18^F]-PI-2620 in vivo and the autoradiography observations of a low binding affinity of this tracer for tau inclusions predominantly made of 4-repeat tau in non-AD tauopathies—similar to that of Flortaucipir and MK-6240—could be due to the removal of some weaker specific PI-2620 binding by the ethanol washing steps that are routinely used in autoradiography protocols to remove unbound radiotracer. Thus, we conducted ethanol-free parallel autoradiographic experiments that yielded identical results of low binding affinity of the three tracers to 4R tau aggregates and allowed us to convincingly rule out this possibility. Moreover, [^18^F]-Flortaucipir, [^18^F]-MK-6240 and [^18^F]-PI-2620 binding signals could all be effectively blocked by competing concentrations of the other unlabeled compounds, providing additional evidence in support of a similar binding affinity of the three tracers as detected by autoradiography studies.

One of the main challenges to reliably identify true tracer binding to 4-repeat tau aggregates characteristic of PSP and CBD by in vivo imaging is the consistently reported, although with variable intensity, elevated PET retention of [^18^F]-Flortaucipir, [^18^F]-MK-6240 and also [^18^F]-PI-2620 in the basal ganglia of older adults regardless of their clinical diagnosis [8]**.** This is a common in vivo finding even in healthy elderly volunteers as well as in AD patients whose brains are not expected to harbor tau pathology in that region pointing to off-target retention in “on-target” PSP and CBD regions [8]. Even though it has been speculated that MAO-B binding might be responsible for the non-specific retention across tau PET ligands in basal ganglia, to date, the exact origin of such off-target in vivo signal remains uncertain. Importantly, one of the first-generation tau PET tracers, THK-5351, was found to demonstrate high binding affinity to MAO-B [38], compromising its value as a tau-specific tracer and prompting the need for the development of alternative tau-specific imaging agents. Studies on potential *off*-target binding of [^18^F]-Flortaucipir, [^18^F]-MK-6240 and [^18^F]-PI-2620 to MAO enzymes continue to be relatively scarce and their results are conflicting. Some studies have suggested that Flortaucipir but not MK-6240 or PI-2620 may bind with similar affinity to tau fibrils and MAO-A and/or B enzymes [53, 54] while others, including our own, point to low binding affinity of both Flortaucipir and MK-6240 to MAO enzymes [1, 59]. The discrepancies among the different studies could be the result of the different techniques (e.g., autoradiography assays in tissue slices vs. binding assays in brain homogenates), isotope labeling (e.g., labeling of the tracer with 18-F vs. 3-H), or types of MAO inhibitors used in each case. Furthermore, the possibility exists that some MAO-A inhibitors like harmine that—unlike clorgyline—is a compound with affinity for both MAO-A and DYRK1A (a kinase that phosphorylates tau at multiple AD-related sites and appears in neurofibrillary tangles), may directly compete with tau ligands by binding to DYRK1A in tau tangles rather than through off-target binding to MAO-A [50, 57]. Our present data derived from autoradiography experiments in the presence of competing concentrations of two different MAO-A inhibitors, clorgyline and harmine, and MAO-B inhibitor, deprenyl, seem to strongly favor this possibility. [^18^F]-Flortaucipir, [^18^F]-MK-6240 and [^18^F]-PI-2620 binding signals are only weakly displaced by MAO-B inhibitor deprenyl, not displaced at all MAO-A clorgyline (a selective MAO-A inhibitor) but very robustly displaced by harmine (a compound with affinity for both MAO-A and DYRK1A). Additional future studies are now needed to confirm or refute these observations. Extrapolation of in vitro results to in vivo imaging findings always requires a very prudent interpretation, especially in the setting of demonstrated off-target binding and potential nonspecific retention due to other biological and technical factors like for example different tracer pharmacokinetics. Future imaging-pathological correlation studies of these three tau tracers conducted on material from individuals scanned while alive will very valuable to conduct detailed quantitative assessments for the reliable interpretation of change of tau pathology burden in AD and the relationship with the clinical progression of the disease.

In conclusion, all together our results from the head-to head comparison of Flortaucipir, MK-6240 and PI-2620, as reported by phosphor-screen and high resolution autoradiographic experiments conducted in the same postmortem tissue material representing the wide spectrum of neurodegenerative dementing disorders, show that these three tau PET tracers exhibit a nearly identical binding profile. They all hold promise as potential surrogate markers for the in vivo detection of tau aggregates in AD, while still having various forms of off-target binding that need to be considered when interpreting their in vivo imaging findings. The utility of Flortaucipir, MK-6240 and PI-2620 for the reliable in vivo detection and tracking of tau pathology in non-AD tauopathies, however, appears very limited. None of these three tracers seems to exhibit significant binding to MAO enzymes. Future imaging-pathological correlation studies on postmortem material from patients scanned while alive continue to be key to further assess the utility of each of these three tracers to accurately quantify regional burdens of tau pathology in AD and to assess response to therapeutic interventions aimed at decreasing or stopping the progression of tau aggregation in AD.

## Data Availability

The datasets generated and/or analyzed during the current study are not publicly available but are available from the corresponding author on reasonable request.
